# The liver sinusoidal endothelial cell hyaluronan receptor and its homolog, stabilin-1 – Their roles (known and unknown) in endocytosis

**DOI:** 10.1186/1476-5926-2-S1-S24

**Published:** 2004-01-14

**Authors:** Peter AG McCourt, Berit Hansen, Dmitri Svistuonov, Sophie Johansson, Paola Longati, Kai Schledzewski, Julia Kzhyshkowska, Sergij Goerdt, Staffan Johansson, B–rd Smedsr–d

**Affiliations:** 1 Department of Experimental Pathology, University of Troms–, Norway; 2 Department of Medical Biochemistry & Microbiology, University of Uppsala, Sweden; 3 Department of Dermatology, Ruprecht Karls University of Heidelberg, Mannheim, Germany

## Introduction

Previous studies have elucidated the importance of the hepatic reticuloendothelial system in clearing waste material from blood. In particular, attention has focused on liver sinusoidal endothelial cells (LEC), which endocytose physiological molecules such as hyaluronan (HA), chondroitin sulphate, denatured collagen, N-terminal pro-peptides of types I and III procollagen, lipoproteins and many others. However, the complete catabolic pathways of these molecules, in particular their uptake by LEC, remain poorly understood. This paper outlines the characterization and cloning [1,2] of an integral endocytic receptor for one such pathway, namely the LEC-HA receptor, and its relationship to stabilin-1, a protein found in discontinuous endothelium and possibly involved in angiogenesis [2,3].

## Methods

The purification/functional characterization of the LEC-HA receptor, and its cloning together with stabilin-1 are described elsewhere [1,2].

## Results and Discussion

We have focused on the endocytic LEC-HA receptor, which we cloned (AJ295695; stabilin-2). We have also cloned a homologous protein with 41% homology but with unknown function, termed stabilin-1 (AJ275213) (2). The normal distribution of stabilin-1 is restricted to the sites of HA removal, i.e., the sinusoidal EC of liver, spleen and lymph nodes [3].

The LEC-HA receptor also has affinity for N-terminal pro-peptides of type I procollagen and formaldehyde treated BSA, both scavenger receptor (SR) ligands. This protein may thus represent a new member of the SR family, a group of proteins shown to play a key role in the uptake of modified low-density lipoproteins leading to the formation of atherosclerotic plaques. We have therefore coined the name HA/scavenger receptor (HA/S-R) to describe its activity [1], but it is also known as "stabilin-2" due to its homology to stabilin-1 [2].

HA/S-R endocytoses AGEs [4], products of non-enzymatic protein glycosylation, a process that occurs especially in the blood of diabetics. The resulting AGEs cause complications through the formation of AGE-amyloids in the vasculature. Our antibody to HA/S-R inhibits LEC uptake of AGE by 50% suggesting that this receptor is vital for AGE clearance. Furthermore, AGE:HA/S-R ligation condemns the receptor to degradation, instead of the normal recirculation to the cell surface [4]. It is therefore possible that this process leads to depletion of liver SEC endocytic capacity, and thus pathological accumulation of AGEs elsewhere.

The role of stabilin-1 is less clear. In cultured LEC, stabilin-1 is restricted to intracellular electron-dense vesicles, and does not come to the cell surface when expressed in various cell lines, where it is restricted to granular cytoplasmic structures. This is consistent with the presence of intracellular targeting domains in stabilin-1, suggesting an intriguing role for this protein. Adding to the mystery, recombinant stabilin-1 does not bind HA despite its similarities to HA/S-R.

The similar structures of HA/S-R and stabilin-1, and their co-distribution in the liver suggest that their roles may somehow be entwined, and may add a new dimension to the catabolic pathways used by LEC to rapidly remove and degrade soluble waste molecules from the circulation.
